# Hepatoid carcinoma colliding with a liposarcoma of the left colon serosa presenting as an abdominal mass

**DOI:** 10.1186/1477-7819-5-42

**Published:** 2007-04-22

**Authors:** Michele Orditura, Eva Lieto, Francesca Ferraraccio, Giuseppe De Cataldis, Teresa Troiani, Paolo Castellano, Giuseppe Catalano, Fortunato Ciardiello, Gennaro Galizia, Ferdinando De Vita

**Affiliations:** 1 Dipartimento Medico-Chirurgico di Internistica Clinica e Sperimentale "F. Magrassi – A. Lanzara" Naples, Italy; 2 Cattedra di Oncologia Medica, Seconda Università degli Studi di Napoli °Ospedale "Da Procida", Salerno, Naples, Italy; 3 Cattedra di Chirurgia Generale ed Epatobiliare, Seconda Università degli Studi di Napoli Ospedale "Da Procida", Salerno, Naples, Italy; 4 Cattedra di Anatomia e Istologia Patologica – Seconda Università degli Studi di Napoli °Ospedale "Da Procida", Salerno, Naples, Italy; 5 Department of Clinical and Experimental Medicine, Second University of Naples School of Medicine, Naples, Italy c/o II Policlinico Via S. Pansini, 5 80131, Naples, Italy

## Abstract

**Background:**

Hepatoid adenocarcinoma (HAC) is a peculiar type of extrahepatic adenocarcinoma generally characterized by adenocarcinomatous and hepatocellular carcinoma (HCC)-like foci. Stomach is the most frequent site where hepatoid adenocarcinoma occurs, although it has been described in many other organs. On the other side, liposarcoma is a rare, malignant tumor that develops from fat cells.

**Case presentation:**

We describe here a case of hepatoid carcinoma in collision with a liposarcoma of the left colon serosa in a 71-year-old man. It presented as an abdominal mass involving several organs, falsely mimicking metastatic colonic adenocarcinoma. Recognition of this entity was evident on microscopic evaluation following surgery. The patient had an objective response following liposomal antracycline chemotherapy, with a 3-year overall survival.

**Conclusion:**

To our knowledge, this is the first case of a hepatoid tumor colliding with a liposarcoma of the left colon serosa reported to date.

## Background

Hepatoid adenocarcinoma (HAC) is a type of extrahepatic adenocarcinoma, which shows a striking morphologic similarity to hepatocellular carcinoma. It is generally characterized by adenocarcinomatous and hepatocellular carcinoma (HCC)-like foci. This variant of adenocarcinoma has been demonstrated to be an alpha-fetoprotein (AFP)-producing carcinoma arising in extra hepatic organs, and it mimics hepatocellular carcinoma in morphological and functional terms.

In most instances, hepatoid carcinoma has been found in the stomach [1,2], but it also occurs in many other organs. Recently, HAC occurrence has been reported in such organs as ileum, gallbladder [3], ovary [4,5], pancreas [6,7], lung [8,9], renal pelvis [10], uterus [11], and urinary bladder [12].

A prompt and accurate diagnosis of hepatoid adenocarcinoma is important because prognosis is very poor compared with that of both common types of adenocarcinoma [13] and AFP-producing adenocarcinomas [14].

Liposarcoma is a rare, malignant tumor that develops from fat cells. It is the most common soft tissue sarcoma and accounts for 20% of all malignant mesenchymal tumors. This tumor typically appears in the deep fat tissues of the thigh or abdomen in people aged 50 to 70. Prognosis varies depending on site of origin, type of cancer cell, tumor size, depth, and proximity to lymph nodes. Metastases are common and the 5-year survival rate for a deep and high-grade liposarcoma is less than 50%. This paper describes the unusual occurrence of a hepatoid carcinoma colliding with a liposarcoma.

## Case presentation

A 71-year-old man was admitted because of abdominal pain in January 2003. His past medical history was significant for a monoclonal gammopathy and surgery for hip prosthesis 2 years before the current admission. At another hospital an abdominal computed tomography (CT) scan had revealed a left-sided mass (10 × 9 cm) involving the diaphragm, spleen, and, from the outside, the left colon flexure. Tumor marker (CEA and CA19-9) serum levels were normal, with the exception of serum AFP, which was markedly elevated (44074.6 ng/ml). However, no CT evidence of primary hepatocellular carcinoma was found. Endoscopy of the upper and lower gastrointestinal tract did not show any primary tumor. A FNAB was performed and cytologic analysis was consistent with a moderately differentiated adenocarcinoma.

Since surgery was deemed not to be feasible, 5-fluorouracil and oxaliplatin-based chemotherapy was administered for a total of 6 cycles, resulting in stable disease.

**Figure 1:**
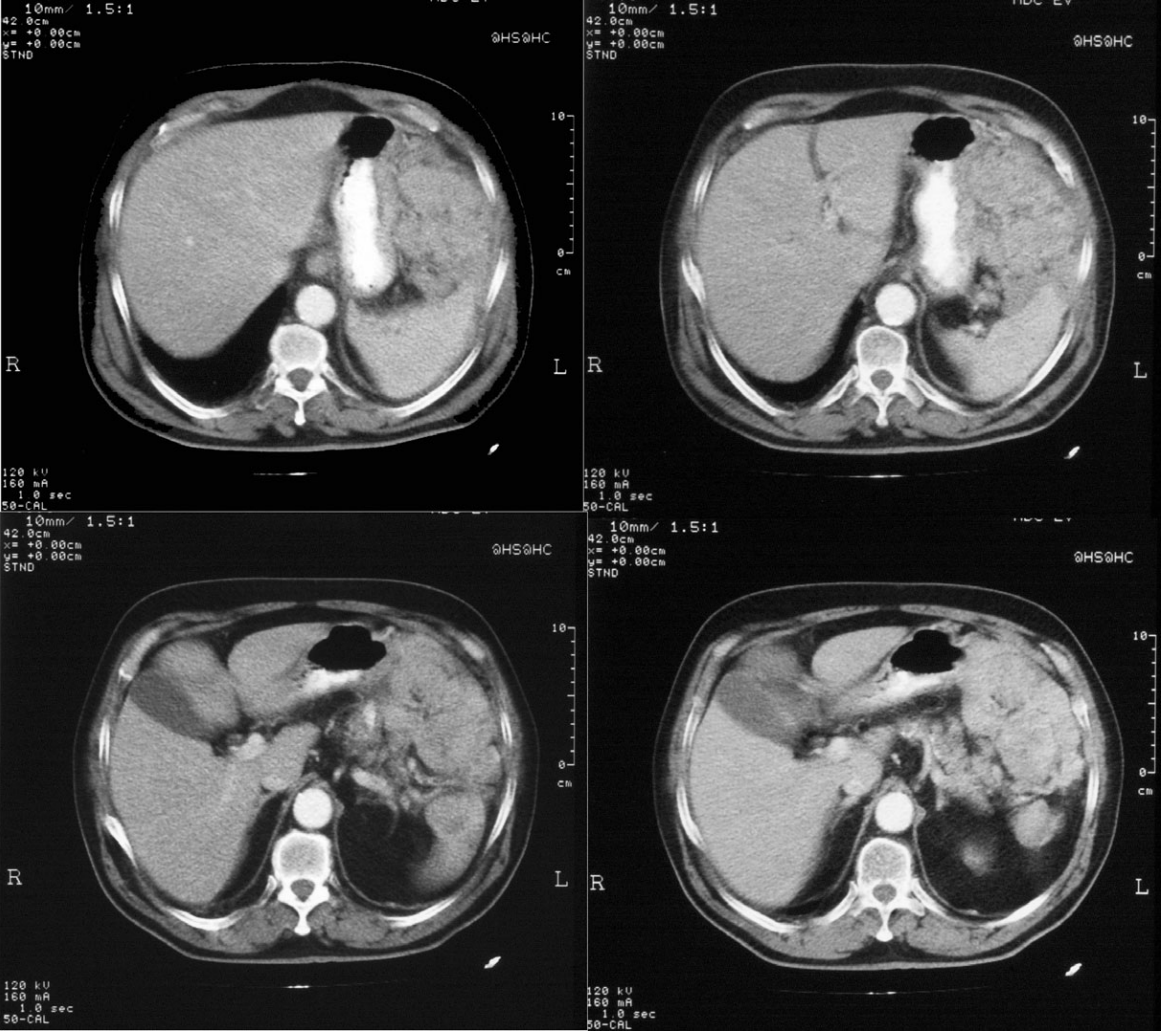
CT scan before the treatment showing the mass lesion.

**Figure 2:**
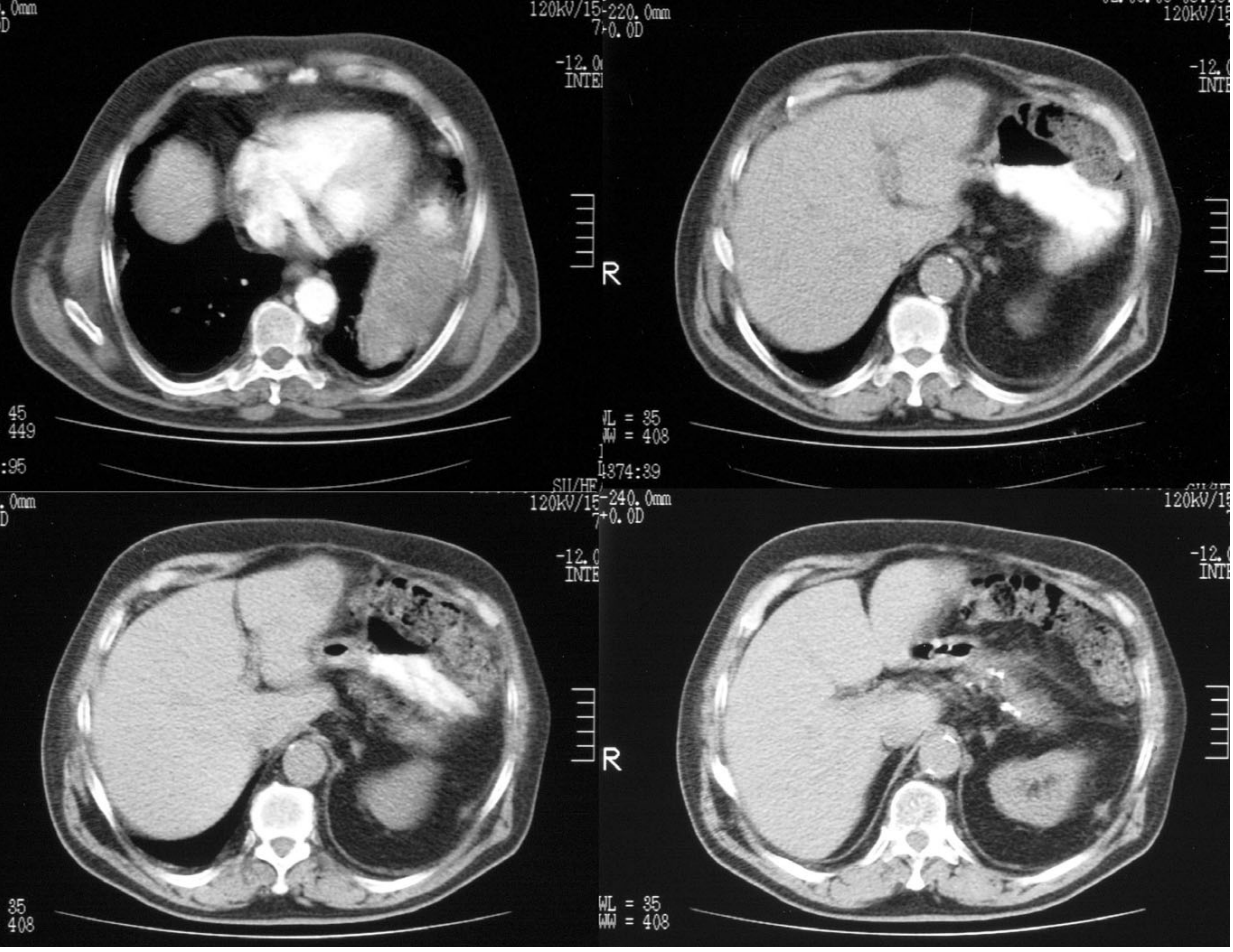
CT scan after the treatment.

As the abdominal mass was shown to have slightly shrunk (Figure 1), a laparotomy was scheduled with the aim to perform a radical operation in July 2003. Unfortunately, along with spleen and left colon, the tumor was found to infiltrate a large part of the left diaphragm thus making it difficult to remove the entire mass. A segmental resection of the left colon (left transverse colon, splenic flexure, and proximal part of the descendent colon) along with splenectomy was performed. A small part of the tumor was left in place (figure 2).

**Figure 3:**
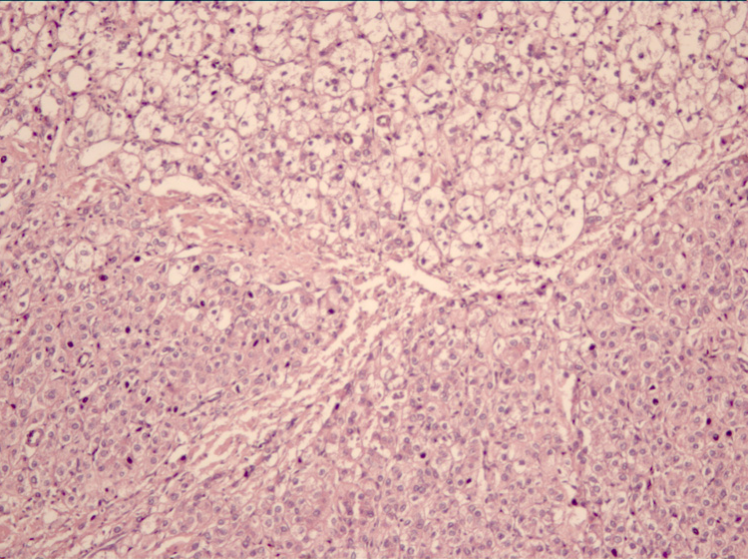
Photomicrograph Showing collision of hepatoid carcinoma (left-up corner) and liposarcoma (left down corner) (H&E × 100 magnification).

The gross appearance of the operated specimen was that of a lobulated, compact, yellow-greenish mass immersed in an adipose-like stromal tissue. Included in the pathological evaluation were also the spleen (12.5 × 6.0 cm), a 7-cm intestinal tract, and tissue samples from colon serosa (6.0 cm), omentum (19 × 18 cm), diaphragm (4.5 × 3.0 cm), peritoneum (8.5 × 3.5 cm), transverse mesocolon (2.5 × 2.0 cm), and pancreatic tail lymph nodes. The spleen had an irregular surface due to the presence of multiple, small gray-whitish nodules, which were also observed in the context of the other sampled tissues. The maximum diameter of the nodules was 2.5 cm. Microscopically, each nodule appeared to be made of single or multiple layers of cells, sometimes with branchlike divisions or in islet-like configuration. Cells in the nodules had polygonal appearance, acidophilic cytoplasm, and large, single or multiple hyperchromatic nuclei, often with vacuolization. These cells stained positive for AFP on immunohistochemistry (Figure 3). In addition, features of well-differentiated liposarcoma were found on colon serosa. In particular, lobules of adipocytes with large and vacuolized cytoplasm were observed. At higher magnification, interspersed lipoblasts with large, hyperchromatic nuclei could be identified. On immunohistochemistry, lipoma-like cells stained positive for S100, vimentin and calretinin but not for p53, p21 WaF1, and c-kit, indicating a well-differentiated liposarcoma.

Finally, a collision zone between the two tumors was identified, consisting of a large area infiltrated by islets or layers of AFP-positive, acidophilic, polygonal cells in contiguity and continuity with islets of lipoblast-rich fat tissue (well-differentiated liposarcoma).

The serum AFP level decreased after surgery (9000 ng/ml). The patient received first line chemotherapy with liposomal doxorubicin for 8 cycles resulting in disease control for 2 years. The patient then had hepatic metastases and died 3 years after the first diagnosis.

## Discussion

The unusual presentation of two entirely different primary malignancies in close proximity to each other is defined as a "collision tumor". To our knowledge, this is the first case of a hepatoid tumor colliding with a liposarcoma of the left colon serosa reported to date. This collision tumor presented as an abdominal mass with extensive involvement of abdominal organs, thus falsely mimicking metastatic dedifferentiated colonic adenocarcinoma. In the absence of a primary identifiable liver disease, this was consistent with a hepatoid carcinoma arising from the peritoneum. The dual nature of the mass was revealed on histologic analysis; it has to be stressed that the two tumors collided at a clear boundary with no evidence of transition both grossly and microscopically; in addition, the immunohistochemical staining pattern for AFP was quite different between the two tumors [[2,9] 15]. In cases such this one, it would be interesting to find out whether these are true collision tumors or single neoplasms with divergent differentiation. However, this was beyond the scope of this report in our case because of the need of complex molecular analyses. Finally, it is uncommon for a liposarcoma to respond to antracycline-based chemotherapy, given its chemo-resistance. A further peculiarity of this case was the unusual presentation of the hepatoid carcinoma, which showed itself as an abdominal extracolonic mass, whereas in other reported cases it would arise in such definite organs as the ovary, the lung or the bladder.

## Conclusion

To data, this is the first case of hepatoid carcinoma colliding with a liposarcoma of the left colon serosa. The unusually good response of this tumor to chemotherapy is worth reporting, especially because systemic chemotherapy is considered little or non effective. Although this is a single case report, patient's response shows that liposomial antracycline therapy is possible for this type of tumor.

## Competing interests

The author(s) declare that they have no competing interests.

## Authors' contributions

MO: Primary author in producing and revising the manuscript. TT, GC, FDV: carried out second line chemotherapy and provided data about follow-up of the patient; Helped to draft the manuscript

FC: helped in scrutiny of manuscript. EL, PC, GG: carried out surgical procedure and provided data about surgical procedure. GDC: carried out the first line of chemotherapy. All authors read and approved the final manuscript.
